# Artificial Intelligence in Dermatopathology: New Insights and Perspectives

**DOI:** 10.3390/dermatopathology8030044

**Published:** 2021-09-01

**Authors:** Gerardo Cazzato, Anna Colagrande, Antonietta Cimmino, Francesca Arezzo, Vera Loizzi, Concetta Caporusso, Marco Marangio, Caterina Foti, Paolo Romita, Lucia Lospalluti, Francesco Mazzotta, Sebastiano Cicco, Gennaro Cormio, Teresa Lettini, Leonardo Resta, Angelo Vacca, Giuseppe Ingravallo

**Affiliations:** 1 Section of Pathology, Department of Emergency and Organ Transplantation (DETO), University of Bari Aldo Moro, 70124 Bari, Italy; anna.colagrande@gmail.com (A.C.); micasucci@inwind.it (A.C.); kcaporusso.c@libero.it (C.C.); lettinit@yahoo.com (T.L.); leonardo.resta@uniba.it (L.R.); 2 Section of Ginecology and Obstetrics, Department of Biomedical Sciences and Human Oncology, University of Bari Aldo Moro, 70124 Bari, Italy; francesca.arezzo@uniba.it (F.A.); vera.loizzi@uniba.it (V.L.); gennaro.cormio@uniba.it (G.C.); 3 Section of Informatics, University of Salento, 73100 Lecce, Italy; mmarangio7@gmail.com; 4 Section of Dermatology, Department of Biomedical Science and Human Oncology, University of Bari Aldo Moro, 70124 Bari, Italy; caterina.foti@uniba.it (C.F.); paolo.romita@uniba.it (P.R.); l.lospalluti@gmail.com (L.L.); 5 Pediatric Dermatology and Surgery Outpatients Department, Azienda Sanitaria Locale Barletta-Andria-Trani, 76123 Andria, Italy; f.mazzotta635@gmail.com; 6 Section of Internal Medicine, Department of Biomedical Sciences and Human Oncology, University of Bari Aldo Moro, 70124 Bari, Italy; sebastiano.cicco@uniba.it (S.C.); angelo.vacca@uniba.it (A.V.)

**Keywords:** AI, machine learning, dermatopathology, skin, future

## Abstract

In recent years, an increasing enthusiasm has been observed towards artificial intelligence and machine learning, involving different areas of medicine. Among these, although still in the embryonic stage, the dermatopathological field has also been partially involved, with the attempt to develop and train algorithms that could assist the pathologist in the differential diagnosis of complex melanocytic lesions. In this article, we face this new challenge of the modern era, carry out a review of the literature regarding the state of the art and try to determine promising future perspectives.

## 1. Introduction

In the last two decades, an unprecedented development of information technologies associated with a considerable increase in memory units has allowed giant strides to be made in the futuristic field of artificial intelligence (AI). Although this is the prerogative of the informatics and technological branches, the use of software and new technologies has spread to many different fields of medicine, indeed to practically all branches, including pathological anatomy and, therefore, also the subbranch of dermatopathology [1,2]. For skin diseases that are more easily diagnosed like basal cell carcinoma (BCC), seborrheic keratosis (SK) and dermal nevus, excellent concordance results have been obtained for the first and embryonic convolutional neural networks (CNN). Instead, as was predictable, the difficulty in diagnosing ambiguous lesions, such as Spitz nevi and rare variants of malignant melanoma, together with the lack of interobserver agreement among dermatopathologists, has led to an objective difficulty in training artificial intelligence algorithms as well as those based on machine learning (ML) to a totally reliable, reportable and repeatable level [3,4]. In this review, we address the most recent issues in relation to the application of AI and ML in dermatopathology, discuss the advantages and limitations of the technologies present to date and consider future prospects.

## 2. Materials and Methods

A systematic review was conducted following the Preferred Reporting Items for Systematic Reviews and Meta-Analyses (PRISMA) guidelines. A search of the PubMed, Medline and Web of Science (WoS) databases was performed until 17 July 2021 using the terms “artificial intelligence” OR “machine learning” OR “digital pathology” in combination with “dermatopathology” OR “skin pathology” OR “digital skin pathology”. Only articles in English were selected.

An independent extraction of articles was performed by two investigators according to the inclusion criteria. Disagreement was resolved by discussion between the two review authors.

## 3. Results

**Figure 1:**
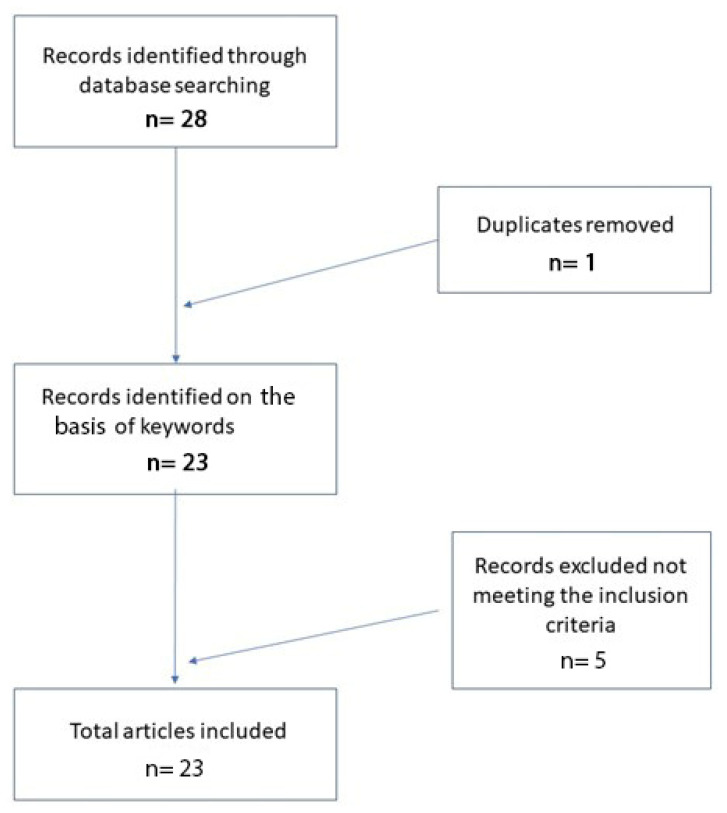
Literature search and article selection.

In total, 28 records were initially identified in the literature search, of which five were related only to the application of AI to clinical dermatology. After screening for eligibility and inclusion criteria, 22 publications were ultimately included (Figure 1). The study and clinical characteristics are summarized in Table 1. The majority of the publications were observational prospective studies (n = 17).

## 4. Discussion

**Figure 2:**
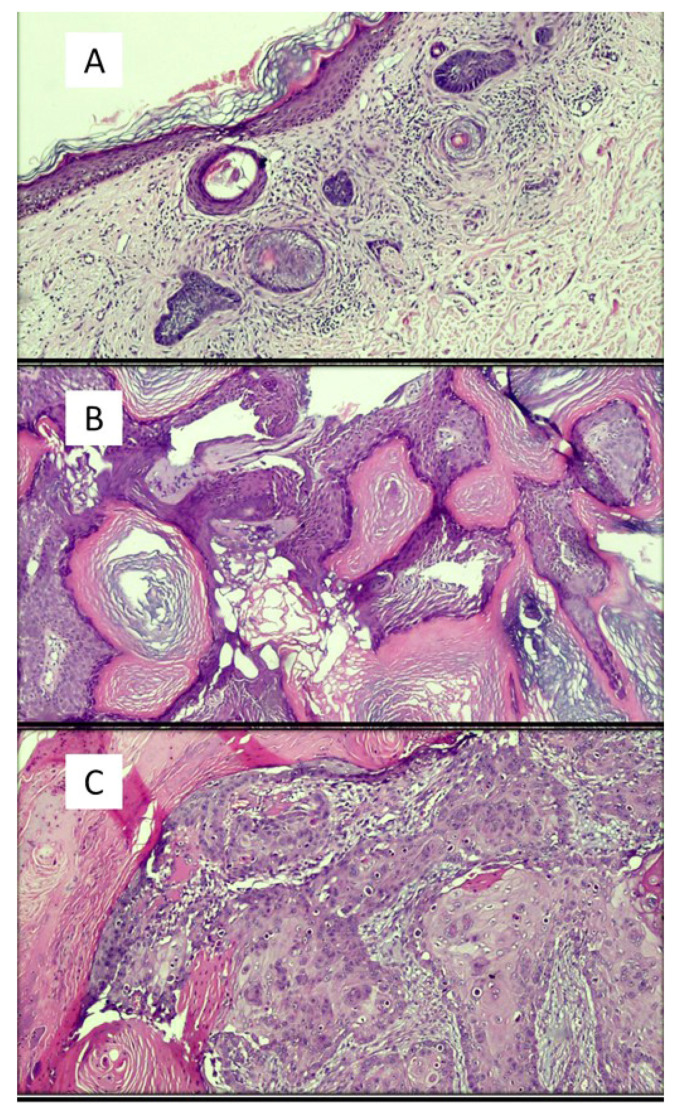

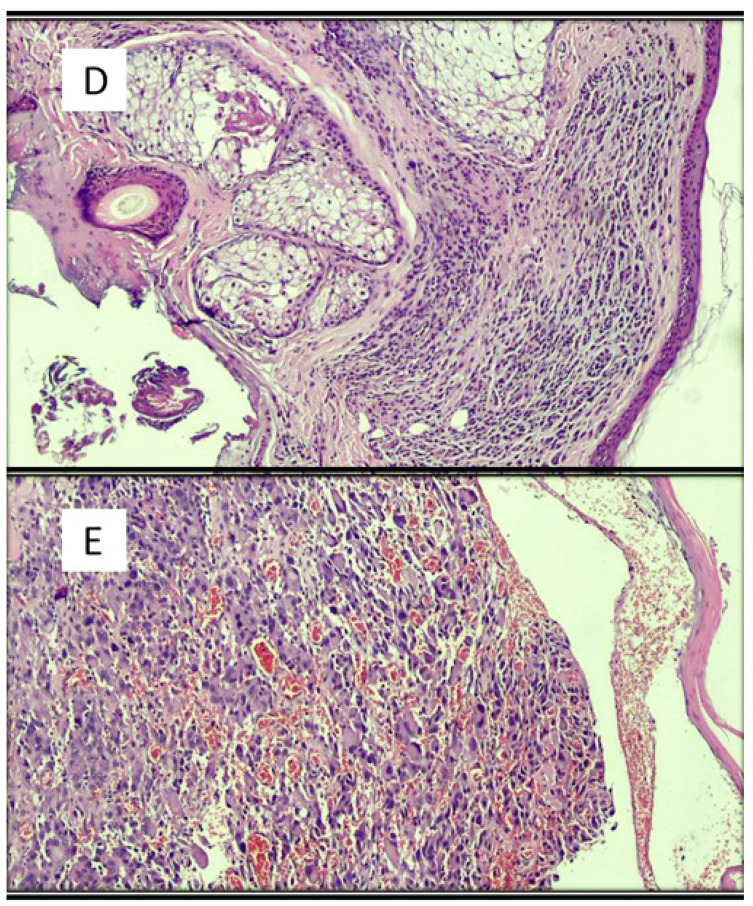
Examples of images of lesions used in various studies in the literature. (**A**) Basal cell carcinoma, superficial variant (hematoxylin–eosin, original magnification: 4×). (**B**) Seborrheic keratosis, hyperkeratotic variant (hematoxylin–eosin, original magnification: 10×). (**C**) Squamous cell carcinoma (hematoxylin–eosin, original magnification: 20×). (**D**) Intradermic nevus (hematoxylin–eosin, original magnification: 10×). (**E**) Amelanotic malignant melanoma (hematoxylin–eosin, original magnification: 20×).

Artificial intelligence applied to pathological anatomy [5] has attracted a particular interest from pathologists and, in more detail, also from dermatopathologists. Increasing attention is being paid to the applications of AI and ML in the diagnosis of simple or more complex skin lesions, and the training of AI algorithms is gathering increasing feedback from the scientific community [4,5,6,7]. In a recent paper by Sam Polesie et al. [8], an attempt was made to understand the degree of perception, attitude and knowledge of AI in general and then as applied to dermatology and dermatopathology. An anonymous, voluntary online survey was prepared and distributed to pathologists who regularly analyze dermatopathology slides/images. The survey consisted of 39 questions divided into five sections: (1) AI as a topic in pathology, (2) previous exposure to AI as a topic in general, (3) applications of AI in dermatopathology, (4) feelings and attitudes towards AI and (5) knowledge of technologies and self-reported demographics. In total, 718 people from 91 countries responded to this survey (64.1% of them women). While 81.5% of the respondents were aware of AI as an emerging topic in pathology, only 18.8% had a good or excellent knowledge of AI. In terms of diagnosis classifications, 42.6% saw a strong or very strong potential for automatic suggestion of the possible skin cancer diagnosis. The corresponding figure for inflammatory skin diseases was 23.0%. For specific applications, the highest potential was considered to be for automatic detection of mitosis (79.2%) and tumor margins (62.1%) and for the evaluation of immunostaining (62.7%). The potential for automatic suggestion of immunostaining (37.6%) and genetic panels (48.3%) was seen as lower. This study highlighted that respondent age did not affect the general attitude towards AI. Only 6.0% of the respondents agreed or firmly agreed that human pathologists will be replaced by AI in the near future. Among the whole group, 72.3% agreed or firmly agreed that AI will improve dermatopathology and 84.1% think AI should be part of medical education. All this demonstrates profound recent changes in the very perception of AI and ML and that an increasing number of applications of these is occurring in various medical fields. For example, in some studies [9,10], artificial intelligence algorithms match or outperform doctors in disease detection related to medical imaging. Additionally, the use of AI has been facilitated by the availability of affordable high-speed Internet, new computing power and secure cloud storage to manage and share datasets. Therefore, it has been possible to make these algorithms scalable on multiple devices, platforms and operating systems, transforming them into modern medical tools [11]. The paper by Esteva et al. [12] applied a deep learning algorithm to a combined skin dataset of 129,450 clinical and dermoscopic images consisting of 2032 different skin lesions. They compared the performance of a deep learning method with that of 21 board-certified dermatologists for the classification and differentiation of carcinomas versus benign seborrheic keratoses and of melanomas versus benign nevi. The AI performance was shown to be on a par with the dermatologists’ performance for skin cancer classification. Deep learning solutions have been successful in the field of digital pathology with whole-slide imaging (WSI). Examples of histopathological images of skin lesions are shown in Figure 2.

Hekler et al. [13] analyzed 695 lesions previously classified by an expert histopathologist according to the guidelines (of which 350 were nevi and 345 were melanomas). Hematoxylin and eosin (H&E)-stained slides of these lesions were scanned using a slide scanner and then randomly cut out; 595 of the resulting images were used to train a convolutional neural network. The additional 100 sections of H&E images were used to test the CNN results against the original class labels. The authors reported a discrepancy with the histopathologist of 18% for melanoma (95% confidence interval (CI): 7.4–28.6%), 20% for nevi (95% CI: 8.9–31.1%) and 19% for the full set of images (95% CI: 11.3–26.7%). These data were held to show that even in the worst case, the CNN mismatch was more or less the same as the mismatch among human pathologists reported in the literature. In addition, despite the greatly reduced amount of data, time required for diagnosis and costs compared to the pathologist, CNNs had a comparable archive performance. Therefore, it was concluded that CNNs can offer a valid diagnostic assistance tool in the hands of a dermatopathologist.

Jiang et al. [14] aimed to develop deep neural network structures for accurate BCC recognition and segmentation based on microscopic ocular images (MOI) acquired by smartphones. To do this, they collected a total of 8046 MOIs, 6610 of which had binary classification labels, while the other 1436 had pixel-by-pixel annotations. Meanwhile, 128 WSIs were collected for comparison. Two deep learning frameworks were created. The “waterfall” framework had a classification model for identifying difficult cases (images with low prediction confidence) and a segmentation model for further in-depth analysis of difficult cases. The “segmentation” framework directly segmented and categorized all images. These authors developed two deep learning frameworks for the recognition of BCCs featuring high sensitivity and specificity, thus inaugurating a valid way to implement their use in clinical diagnosis of images acquired by smartphones.

Cruz-Roa et al. [15] used a deep learning architecture to discriminate between BCC and normal tissue models in 1417 images from 308 regions of interest (ROIs) of skin histopathology images. They compared the deep learning method to traditional machine learning with feature descriptors, including feature pack, canonical and Haar wavelet transformation. The deep learning architecture proved superior to traditional approaches, reaching 89.4% in F-measure and 91.4% in balanced accuracy.

Table 1 summarizes other studies present in the literature and not mentioned in the review.

Peizhen et al. [21] built a multicenter database of 2241 digital images of whole slides of 1321 patients from 2008 to 2018. They trained both ResNet50 and Vgg19 using over 9.95 million patches by transferring learning and test performance using two types of critical classifications: melanoma malignant versus benign nevi in separate and mixed magnification, and distinguishing nevi at maximum magnification. CNNs achieved a superior performance in both activities, demonstrating that artificial intelligence is capable of classifying skin cancer in histopathological image analysis. To make the classifications reasonable, the visualization of CNN representations was also used to identify cells between melanoma and nevi. Regions of interest (ROIs) were also localized, which was significantly useful, offering pathologists greater support for the correct diagnosis. Although the development of ML-based AI is spreading in dermatopathology, we are still quite far from its application in clinical routine because, even when compared to the algorithms applied in dermoscopy, there is less sensitivity and specificity and hence less accuracy. More specifically, some histological lesions closely mimic other types of neoplasms, such as skin adnexal lesions, which can require differential diagnosis with BCC, SCC, KS or melanoma [13]. Furthermore, despite rather promising values, these algorithms are not able to diagnose a malignant lesion (for example, melanoma) in all cases, thus making their use unacceptable without human control [13,14,15,20,21,22,23].

## 5. Conclusions

New technologies are making possible what was considered futuristic only a few years ago. AI and ML as machine learning models have shown that they can learn from their mistakes, self-correct and thus be able to provide valid support to dermatopathologists. We have not reached such levels as to allow a complete “replacement” of pathologists, but they can provide a good aid in histopathological diagnosis. On the other hand, the dermatopathological diagnosis consists of a whole corollary of clinical information, such as the age of the patient, topography of the lesion, dermoscopic characteristics, color variations, ulceration, that a neural network is not able to take into account, being based fundamentally on image discrimination. It is also important to underline how much dermatopathology is a terrain of diagnostic debate among the pathologists themselves and how much the atypical lesions that are not easily framed in defined criteria (such as particular forms of Spitz nevi, atypical nevi / Spitz tumors, melanocytic proliferations of uncertain clinical significance such as SAMPUS and MELTUMP and variants of melanoma such as Spitzoid melanoma) are far from finding agreement in “real life”, affecting all this, even in the difficulty in being able to train a convolutional neural network in certain recognition of an entity. Finally, we must not overlook the fact that any artificial intelligence algorithm is trained by a “human being” and, therefore, will always suffer from “involvement” on the part of the pathologist.

This confirms the need for new studies and discoveries in areas that have not yet been sufficiently explored, such as dermatopathological diagnosis served by AI.

## Figures and Tables

**Table 1:** Other studies present in the literature besides those analyzed in the Discussion section of this work.

Authors	Years	Type of AI	Results	Strengths	Limits
Potter et al. [16]	1987	Interactive computer program	Concordance, 91.8% Disagreement, 4.8%	Concordance and possibility of integration with patient clinical data	Disagreement and little memory space
Crowlet R. et al. [17]	2003	Traditional intelligent tutoring system	Possibility of learning rather easily	Positive feedback	Clear prototypical schemes are indispensable
Joset Feit et al. [18]	2005	Hypertext atlas of dermatopathology	A collection of about 3200 dermatopathological images	Continuous updating	/
Payne et al. [19]	2009	Intelligent tutoring system	Tutoring made it possible to implement the training of learners	Ability to learn from mistakes	Greater difficulties in tutoring related to superficial perivascular dermatitis
Olsen et al. [20]	2018	Deep learning algorithms	The artificial intelligence system accurately classified 123/124 (99.45%) BCCs (nodular), 113/114 (99.4%) dermal nevi and 123/123 (100%) seborrheic keratoses	Concordance	Difficulty in presenting artifacts, poor coloring
